# Pharmacokinetics, Urinary Excretion, and Pharmaco-Metabolomic Study of Tebipenem Pivoxil Granules After Single Escalating Oral Dose in Healthy Chinese Volunteers

**DOI:** 10.3389/fphar.2021.696165

**Published:** 2021-07-13

**Authors:** Zeyun Li, Mei Su, Weiyan Cheng, Jueyu Xia, Shuaibing Liu, Ruijuan Liu, Suke Sun, Luyao Feng, Xueya Zhu, Xiaojian Zhang, Xin Tian, Lingbo Qu

**Affiliations:** ^1^ Department of Pharmacy, The First Affiliated Hospital of Zhengzhou University, Zhengzhou, China; ^2^ Henan Key Laboratory of Precision Clinical Pharmacy, Zhengzhou University, Zhengzhou, China; ^3^ Jiangsu Carephar Pharmaceutical Co., Ltd, Nanjing, China; ^4^ College of Chemistry and Molecular Engineering, Zhengzhou University, Zhengzhou, China

**Keywords:** tebipenem pivoxil, pharmacokinetics, urinary excretion, metabolites, pharmaco-metabolomics

## Abstract

Tebipenem pivoxil (TBPM-PI), an oral carbapenem antibiotic, has shown special advantages in pediatric infections and was in urgent need in China. Although pharmacokinetics, urinary excretion, and metabolite information of its active form tebipenem (TBPM) has been reported, ethnic differences may exist among the Chinese and Japanese population. By now, no systematic pharmacokinetics, urinary excretion, metabolites, or safety information has been revealed to the Chinese population. The purpose of the present work was to investigate abovementioned information of TBPM-PI granules after oral single ascending doses of 100, 200, and 400 mg in Chinese volunteers. Based on the pharmacokinetic study, the urine pharmaco-metabolomic analysis was conducted to reveal metabolomic interruptions and metabolite information. The study design was a single-center, open-label, randomized, single-dose pharmacokinetic study of 36 healthy volunteers (with half of them being male and the other half female). Time to maximum concentration (*T*_max_) was reached at 0.50, 0.50, or 0.67 h for 100, 200, or 400 mg, respectively. The linear pharmacokinetic characteristic of maximum plasma concentration (*C*_max_) was detected over 100–200 mg. The area under the concentration time curve (*AUC*) was proportional to the dose in the range of 100–400 mg. The maximum urinary excretion rate was detected at 0–1 or 1–2 h for dose of 100 or 200–400 mg. Cumulative amount of TBPM excreted in urine by 24 h accounted up to 90, 95, and 80% of dose administered for three groups, respectively. The pharmaco-metabolomic analysis revealed urine metabolic trajectory of deviation at 0–1 or 1–2 h and gradually regressing back to the pre-dose group at the following time periods. Urine metabolites from M1 to M4 were identified, indicating ethnic difference in metabolites among the Chinese or Japanese population. The current work proved safety and tolerance of single-dose administration of oral TBPM-PI in Chinese healthy volunteers over doses of 100–400 mg. All these results provide pharmacokinetics, urine excretion, urine metabolomics, urine metabolites, and safety information in healthy Chinese volunteers after oral single ascending doses of TBPM-PI, benefitting further development and clinical utilities.

## Introduction

Tebipenem pivoxil (TBPM-PI) granules were the first oral carbapenem antibiotic approved in Japan in 2009, and it has been widely used for the treatment of pneumonia, otitis media, and sinusitis in pediatric patients in Japan (Kobayashi et al., 2005; Yao et al., 2016; Jain et al., 2018). Due to its insusceptibility to drug resistance, TBPM-PI granules have shown special advantages in pediatric infections (McEntee et al., 2019; Yao, et al., 2016). Drug resistance in pediatric infections has been frequently reported in China in the recent years (Chen et al., 2018; Ai et al., 2020). TBPM-PI has been proven valuable for treatment of drug resistance bacteria reported in China and is in urgent need to be introduced into clinical application.

As an inactive prodrug, TBPM-PI was required to metabolize to its active form of tebipenem (TBPM), to exert antibacterial effects (Jain et al., 2018). TBPM-PI was absorbed by multiple intestinal transport routes (including uptake transporters OATP) and then metabolized to TBPM or its metabolites by drug-metabolizing enzymes (including epoxide hydrolase and renal dehydropeptidase-I) (Jain, et al., 2018; Kato et al., 2010). Main metabolites observed in TBPM were its open-ring forms, which were suspected with carbapenem allergies due to a structural similarity of a beta-lactam ring (Sato et al., 2008; Lee and Bradley, 2019). TBPM was mainly excreted in urine, with cumulative urinary excretion of 60–70% by 24 h (80% if open-ring metabolites are included) (Nakashima et al., 2009b). The abovementioned aspects may contribute to interethnic differences of pharmacokinetics, renal elimination, and metabolites, which may influence Chinese patient’s clinical outcome and warrant further research.

Although pharmacokinetics and renal elimination of TBPM have been well characterized in the Japanese population (*T*_max_ 0.55–0.90 h, t_1/2_ 0.40–0.91 h, *C*_max_ or *AUC*_0-∞_ increasing dose proportionally over 25–500 mg, up to 70% of protype recovered in urine by 24 h) (Nakashima et al., 2009a), pharmacokinetic profiles, urine excretion and metabolite information, and safety profile of TBPM in Chinese population have rarely been revealed (Xue et al., 2019). As a result, pharmacokinetics, urine excretion, and safety profiles were imperative to elucidate abovementioned information in Chinese population.

Generally, pharmacokinetics data were collected in the pharmacokinetic study, leaving a large amount of other information including metabolic interventions and metabolites of drugs unexploited. Pharmaco-metabolomics, as a powerful tool to fully reveal metabolic interventions of drugs, has been used to predict drug metabolism, pharmacokinetics, drug safety, and drug efficacy (Winnike et al., 2010; Blasco et al., 2018). However, it has been seldom applied in the clinical pharmacokinetic study in healthy volunteers. In the current work, the pharmaco-metabolomic analysis was carried out for urine samples, to investigate drug interruption of metabolic profiles and to reveal urine metabolites of TBPM-PI. As far as we know, the current work was the first application of pharmaco-metabolomics in the pharmacokinetic study of healthy volunteers, which may further provide safety, elimination, and metabolite information, benefiting drug clinical research.

The present study aimed at providing pharmacokinetics, urinary excretion, urinary metabolomics, and metabolite information of TBPM-PI in the Chinese population after oral administration of TBPM-PI granules. According to the guideline for clinical pharmacokinetic study of chemical drugs ([H]GCL1-2) and previous reports, 36 health Chinese volunteers (three groups) were recruited and randomly dosed TBPM-PI of 100, 200, 400 mg (low, medium, and high doses). Pharmacokinetics and urinary excretion of TBPM were investigated by the LC-MS/MS method. A pharmaco-metabolomic study of accumulated urine was conducted to provide metabolite and elimination information by UPLC-Q-TOF. The methods employed in current research were well developed. In general, pharmacokinetics, urinary excretion, pharmaco-metabolomics, metabolite and safety information of TBPM-PI were revealed for the first time after single escalating oral doses in healthy Chinese volunteers.

## Materials and Methods

### Chemicals and Reagents

TBPM (Batch No. 2470-052A2, purity 98.9%) was obtained from TLC Pharmaceutical Standards Ltd. (Newmarket, Canada). Ceftizoxime (Batch No.: 130,504–201503, purity 98.5%) was purchased from the National Institutes for Food and Drug Control (Beijing, China) and used as an internal standard (IS). HPLC-grade methanol and acetonitrile were purchased from TEDIA (Fairfield, CT, United States). Formic acid (HPLC grade) was obtained from Fisher Scientific (Waltham, MA, United States). Ammonium acetate (HPLC grade) was purchased from Beijing Reagent Company (Beijing, China). Distilled water was filtered through the Milli-Q system (Millipore, MA, United States).

### Subject Recruitment

The study was approved by the Institutional Review Board of the First Affiliated Hospital of Zhengzhou University (approval number, Drug-2017-38) and registered at http://www.chinadrugtrials.org.cn (registration number CTR20181402). Only healthy young male and female subjects aged over 18 years with body mass index (BMI) of 19–26 kg/m^2^ were included. All subjects signed a consent form and were in good health as confirmed by their medical history and physical examination. Subjects with diagnosed digestive, urinary, cardiovascular, circulatory, respiratory, neuropsychiatric, endocrine, and metabolic diseases were ineligible for enrollment. Subjects were also excluded if they had drug abuse or if they tested positive in drug screening; allergic reactions to carbapenems, penicillins, or cephalosporins; recent administration of any other drugs (past 14 days); daily smoking over five cigarettes; or abnormalities in 12-lead electrocardiographic (ECG), blood biochemistry, urine biochemistry, serum virology, or blood clotting function tests. Lactating females or those who tested positive in a pregnancy test were also excluded.

### Study Design

The study was conducted as a single-center, open-label, randomized, single-dose, parallel pharmacokinetic study. TBPM-PI granules (50 mg/bag, batch No. S41-160903) were provided by Jiangsu Carephar Pharma Co., Ltd. (Nanjing, China). The primary objective of the study was to investigate the pharmacokinetic characteristics of TBPM-PI granules at doses of 100, 200, and 400 mg under fasting conditions in healthy Chinese volunteers. Based on the pharmacokinetic study, pharmaco-metabolomic research was conducted to reveal metabolomic interruptions and metabolite information in urine. Prior to study commencement, using a gender-stratified blocked randomization scheme generated by SAS 9.4, 36 study subjects were randomly assigned to three dose groups of TBPM-PI granules. Treatment compliance was 100% as subjects were dosed under direct supervision. Subjects were under constant surveillance by clinical staff during the time they were confined to the research facility, a minimum of 10 h prior to drug administration until permission to leave the clinical research center.

### Sample Collection

Blood samples were withdrawn through an indwelling cannula (placed at the morning of Day 1) 30 min prior to study drug administration and at 16 time points, namely, 5, 10, 15, 20, 30, 40, 50 min, 1, 1.5, 2, 3, 4, 5, 6, 7, and 8 h after administration. These time intervals were considered appropriate with regard to a terminal half-life period (*t*_1/2_) of 0.8 h for TBPM (Xue, et al., 2019). The volume of blood drawn from each time point did not exceed 4 ml. The blood samples were collected in an ice-water bath and centrifuged for 10 min at 2500 *g* at 4 ± 2°C. At least 0.5 ml of the plasma was dispensed into polypropylene tubes and deposited into a −80°C freezer prior to being analyzed.

Urine samples were collected prior to study drug administration and at seven time periods: 0–1, 1–2, 2–4, 4–6, 6–8, 8–12, and 12–24 h after administration. Urine in each time period was mixed, and the volume was recorded. 5 ml of urine were dispensed into polypropylene tubes and deposited into a −80°C freezer prior to being analyzed.

### Pharmacokinetic Analysis

The plasma concentration of TBPM was determined by a LC-MS/MS system which constituted an Agilent 1200 HPLC system (Agilent, United States) and an SCIEX API 4000 mass spectrometer (AB Sciex, Framingham, MA, United States). Plasma samples were processed as follows: 50 μl plasma was added along with 50 µl IS (ceftizoxime, 20.0 μg/ml) and 150 µl of acetonitrile, vortexed for 5 min, and centrifuged (3900 rpm) at 4°C for 10 min. After being diluted twice with water, 3 μl of the final solution was injected into the LC system. TBPM was separated on an XDB-C18 column (1.8 μm, 4.6 × 50 mm, Agilent, United States) with an isocratic elution of 85% mobile phase A (water containing 0.17% formic acid and 8.5 mM ammonium acetate) and 15% mobile phase B (acetonitrile). The flow rate was set at 0.5 ml/min for 0–2.4 min and 0.8 ml/min for 2.41–3.40 min. The column temperature was 40°C. The mass spectrometer was operated using a turbo spray source in the positive multiple reaction monitoring mode (MRM). Quantification was performed using transitions of m/z 384.2→298.1 and m/z 348.1→227.1 for TBPM and IS, respectively. The optimal instrument conditions were set as follows: curtain gas (CUR), nebulizer gas (gas 1), and heater gas (gas 2) were 40, 50, and 50 psi, respectively, and the dwell time (DW) was 300 ms; the turbo spray temperature (TEM) was 500°C; and the ion spray voltage (ISVF) was 5500 V. De-clustering potentials (DPs) were 50 and 63 V for TBPM and IS, respectively; collision energy (CE) was 25 and 29 eV for TBPM and IS, respectively. Analyst 1.6.3 software package (AB SCIEX LLC., Redwood City, CA, United States) was used for chromatography data acquisition and integration.

The analytical method was validated in accordance with the guidelines proposed by the U.S. FDA in the following aspects: selectivity, linearity and lower limit of quantification (LLOQ), accuracy and precision, carryover, matrix effect and extraction recovery, and stability. The pharmacokinetic parameters of TBPM in healthy volunteers were calculated by the noncompartmental analysis using Phoenix WinNonlin version 7.0 (Certara United States, Inc., Princeton, NJ, United States). The following parameters were accessed: *C*_max_, the maximum observed concentration; *T*_max_, the time of observed; *C*_max_; *AUC*_0–t_, calculated by the linear trapezoidal rule; *AUC*_0–∞_, calculated as *AUC*_0–t_ +*C*_t_/*K*_e_, where *C*_t_ was the last measurable concentration and *K*_e_ was the elimination rate constant, estimated from the terminal log decay phase using linear regression; *t*_1/2_ was the elimination half-life, calculated as 0.693/*K*_e_. The gender effect on pharmacokinetics of TBPM was evaluated. A power model (Y = α*(dose)^β^) as described (Kevin et al., 2016) using the modification (Smith et al., 2000) was used to assess the dose proportionality of the PK parameters.

### Urinary Excretion

Urine concentrations of TBPM were determined by the LC-MS/MS method. Briefly, 50 μl of the urine sample and 25 µl IS (ceftizoxime, 20.0 μg/ml) were vortexed for 5 min and centrifuged (3900 rpm) at 4°C for 10 min. After being diluted 15 times with water, 1 μl of the final solution was analyzed. The separation was achieved in an XDB-C18 column (1.8 μm, 4.6 × 50 mm, Agilent, United States) with an isocratic elution of 70% mobile phase A (water containing 0.005% formic acid) and 30% mobile phase B (acetonitrile). The column temperature was 40°C. The flow rate was set at 0.4 ml/min. A triple QuadTM 5500 Mass spectrometer was employed for data acquisition and operated in the positive MRM mode. The MASS conditions were similar as described in the part of “Pharmacokinetic Analysis,” except the following parameters: CUR 35 psi, DW 100 ms, DP 100 V, and CE 30 eV for both TBPM and IS. The established method was fully validated. The urinary excretion rate and percent of dose recovered in urine were calculated by the noncompartmental analysis using Phoenix WinNonlin version 7.0 (Certara United States, Inc., Princeton, NJ, United States).

### Pharmaco-Metabolomic Analysis

Metabolite profiles of time period urine were obtained by an UPLC-ESI-Q-TOF system (AB Sciex, Framinghan, MA, United States). Urine samples were diluted with four times the volume of methanol and centrifuged at 10,000 rpm, 10 min before an injection of 5 µl for analysis. Chromatography separation was achieved on an ACQUITY UPLC HSS T3 column (100 × 2.1 mm i. d., 1.8 μm) maintained at 40°C, with a mobile phase constituting 0.1% formic acid water(A)–acetonitrile (B) and gradient elution as follows: 0–18 min, 1%–50%B; 20–23 min, 100% B; 23.01–26 min, 1% B. The mass data were collected on a SCIEX X500R QTOF mass spectrometer (AB Sciex, Framinghan, MA, United States) coupled with an electrospray ionization interface in positive ion modes (ESI+). SCIEX OS software 1.4 (AB, Milford, MA) was employed for data acquisition and processing. The following parameter settings were used: ISVF of 5500 V, TEM of 600°C, DP of 80 V, CE of 35 V, gas one of 55 psi, gas two of 55 psi, CAD gas of seven psi, and CUR of 35 psi. Nitrogen was kept as the nebulizer and auxiliary gas. TOF MS and TOF MS/MS were scanned with the mass range of m/z 50–1000. A continuous recalibration was carried out for every six samples. In addition, dynamic background subtraction (DBS) and information dependent acquisition (IDA) were used to trigger the acquisition of MS/MS information of low-level constituents. The accurate mass and composition for the precursor ions and fragment ions were analyzed using the Markerview^™^ software (version 4.1, Waters Co., Milford, MA, United States) integrated with the instrument.

Raw MS data were deconvolved and aligned with the following parameters: the retention time (RT) of 0.5–23 min, the mass tolerance of 20 PPM, the ion intensity threshold of 300 counts, and the RT tolerance of 0.1 min. The data were further filtered with the 80% rule (Smilde et al., 2005). Output data were separately imported into SIMCA (version 14.0, Umetrics, Umeå, Sweden) for the multivariate statistical analysis (unit variance scaled). Models of the principal component analysis (PCA) and orthogonal signal correction partial least-square discriminant analysis (OPLS-DA) were applied to investigate metabolic alterations of urine samples after TBPM-PI consumption. The qualities of models were validated by determining *R* ^2^ (goodness of fit parameter) and Q^2^ (goodness of prediction parameter) values. The variation tendency was visualized in the form of score plots, where each point represents a urine sample; the pivotal metabolites were inspected by S-plots, where each coordinate represents one mass-RT feature. Corresponding variables with variable importance in the projection value (VIP) > 1.0 were chosen as candidates for metabolites. The LC-MS peak of TBPM was identified according to the actual mass, MS/MS fragments and RT. Putative identified metabolites were verified by comparing the actual mass and MS^2^ fragmentations, with theory fragmentations obtained by SCIEX OS software.

### Safety Assessment

All subjects participating in the study were included in the safety analysis. Safety was evaluated by continuous observation of adverse events (AEs), monitoring of vital signs, ECG, hematology, biochemistry, and urinalysis at baseline, during the trials at the set time points following drug administration, and at follow-up visits after study completion. All observed or spontaneously reported AEs were recorded, and the severity of AEs was graded according to the National Cancer Institute Common Terminology Criteria for Adverse Events version 4.03.

## Results

**FIGURE 1:**
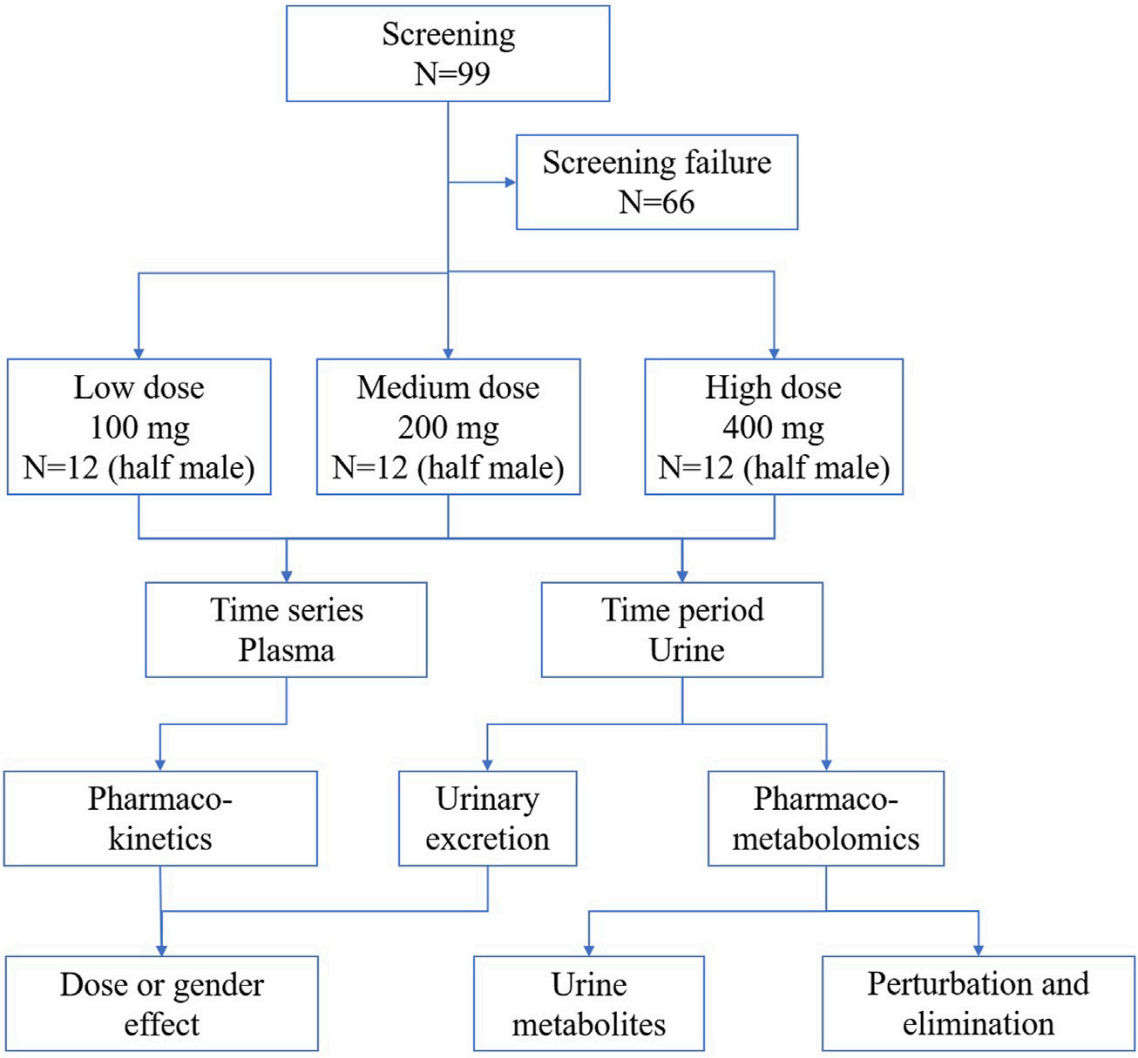
Flowchart of the current work.

Ninety-nine healthy volunteers were screened, of which 36 healthy subjects (with half of them being male and the other half female) were enrolled and randomized to receive TBPM-PI at doses of 100, 200, or 400 mg (12 individuals in each group and half males) in the study. All participants completed the study without important protocol deviations and were included in PK or urine pharmaco-metabolomic analysis. Baseline demographic characteristics for participants in each group are shown in Table 1. Demographic data were similar across treatment groups (mean age, 27.33, 28.76, and 27.17 years; mean BMI, 22.33, 21.79, and 22.17). The flowchart of the current work is shown in Figure 1.

**TABLE 1:** Baseline characteristics of subjects included in the pharmacokinetic analysis population.

	—	TBPM-PI (100 mg, *n* = 12)	TBPM-PI (200 mg, *n* = 12)	TBPM-PI (400 mg, *n* = 12)
Age (years)	Mean ± SD	27.33 ± 3.77	28.67 ± 4.77	27.17 ± 4.28
	Range	23.00–34.00	23.00–36.00	20.00–35.00
Gender	Male/female	6/6	6/6	6/6
Ethnicity	Ethnic Han	12 (100.00%)	12 (100.00%)	12 (100.00%)
Height (cm)	Mean ± SD	166.58 ± 7.66	166.42 ± 6.98	166.13 ± 7.90
	Range	153.50–175.50	157.00–180.50	149.50–180.00
Weight (kg)	Mean ± SD	62.09 ± 8.27	60.58 ± 7.77	61.26 ± 6.61
	Range	50.20–78.40	47.10–72.50	49.40–74.40
BMI (kg/m^2^)	Mean ± SD	22.33 ± 2.18	21.79 ± 1.84	22.17 ± 1.38
	Range	19.20–25.60	19.10–24.80	20.00–24.20

### Pharmacokinetics

**FIGURE 2:**
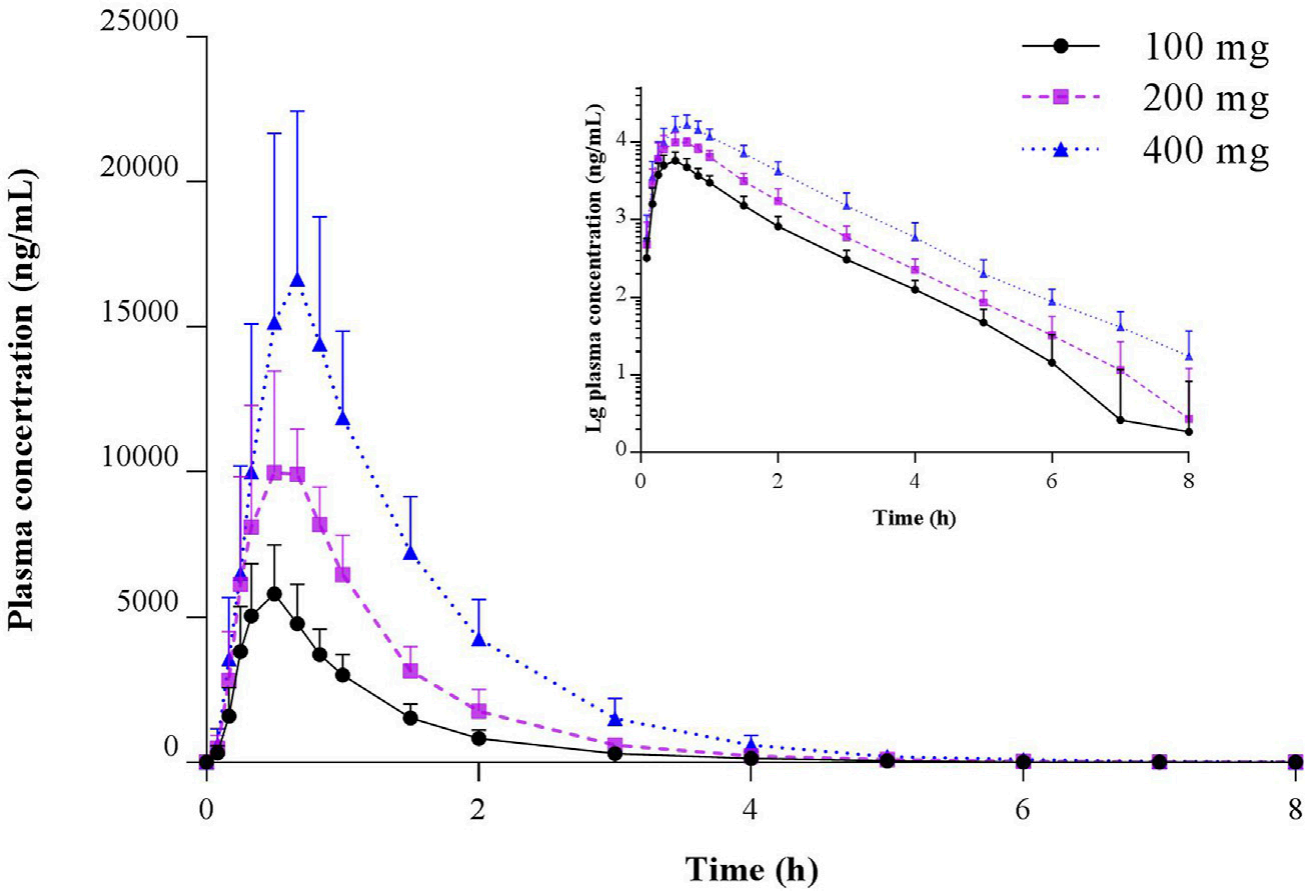
Mean (SD) and semilogarithmic scaled plasma concentration–time profiles of TBPM-PI after a single oral dose of 100, 200, or 400 mg in healthy Chinese subjects (*n* = 12 for each dose).

Pharmacokinetic studies of TBPM in Chinese following oral administration of TBPM-PI granules were successfully conducted. The mean plasma concentration–time profiles of TBPM after a single oral dose of 100, 200, or 400 mg are shown in Figure 2, and the pharmacokinetic parameters are summarized in Table 2.

**TABLE 2:** Pharmacokinetic parameters of TBPM after oral administration at dosage of 100, 200, and 400 mg.

Pharmacokinetic parameters		TBPM-PI (100 mg, *n* = 12)	TBPM-PI (200 mg, *n* = 12)	TBPM-PI (400 mg, *n* = 12)
*C*_max_ (ng/ml)	Mean ± SD	6087.50 ± 1642.15	11099.17 ± 2509.34	17825.00 ± 5753.75
	GM (CV%)	5856.95 (26.98)	10868.83 (22.61)	16993.06 (32.28)
*AUC*_0-t_ (h·ng/m)	Mean ± SD	6364.93 ± 917.09	12483.81 ± 2025.28	23080.77 ± 2668.17
	GM (CV%)	6300.67 (14.41)	12334.50 (16.22)	22932.68 (11.56)
*AUC*_0-∞_(h·ng/ml)	Mean ± SD	6407.34 ± 915.06	12517.70 ± 2024.04	23126.57 ± 2671.22
	GM (CV%)	6343.98 (14.28)	12368.97 (16.17)	22978.55 (11.55)
*T*_max_ (h)	Median (range)	0.50 (0.33, 1.00)	0.50 (0.33, 0.83)	0.67 (0.50, 1.00)
	Mean ± SD	0.50 ± 0.17	0.57 ± 0.13	0.68 ± 0.18
*K*_e_ (h^−1^)	Mean ± SD	0.8963 ± 0.1862	0.9411 ± 0.1445	0.7968 ± 0.7968
	GM (CV%)	0.8699 (20.78)	0.9294 (15.36)	0.7749 (22.82)
*t*_1/2_ (h)	Mean ± SD	0.84 ± 0.34	0.76 ± 0.15	0.92 ± 0.27
	GM (CV%)	0.80 (40.25)	0.75 (19.30)	0.89 (28.8)
*MRT*_0-t_ (h)	Mean ± SD	1.09 ± 0.19	1.13 ± 0.18	1.32 ± 0.24
	GM (CV%)	1.08 (17.12)	1.11 (16.01)	1.30 (18.26)
*MRT*_0-∞_ (h)	Mean ± SD	1.13 ± 0.19	1.14 ± 0.18	1.34 ± 0.24
	GM (CV%)	1.11 (16.92)	1.13 (15.77)	1.32 (18.10)
*CL/F* (ml/h)	Mean ± SD	15932.54 ± 2539.05	16366.41 ± 2697.32	17526.16 ± 2201.04
	GM (CV%)	15762.98 (15.94)	16169.50 (16.48)	17407.54 (12.56)
*Vd/F* (ml)	Mean ± SD	19274.13 ± 8785.59	17709.26 ± 3499.09	23350.63 ± 7184.97
	GM (CV%)	18120.91 (45.58)	17397.71 (19.76)	22465.33 (30.77)

Note: GM, geometric mean; CV%, percent coefficient of variation of geometric mean; *AUC*_0-t_, the area under the concentration–time curve from time zero to the time of last measurable concentration; *AUC*_0-∞_, the area under the concentration–time curve from time zero to time infinity (extrapolated); *C*_max_, the maximum observed concentration; *T*_max_, time of observed *C*_max_; *K*_e_, elimination rate constant; *t*_1/2_, the elimination half-life; *MRT*_0-t,_ the mean residence time under the concentration–time curve from time zero to the time of last nonzero concentration; *MRT*_0-∞,_ the mean residence time under the concentration–time curve from time zero to time infinity (extrapolated); *CL/F*, plasma clearance; *Vd/F,* apparent volume of distribution.

The current work revealed that *C*_max_, *AUC*_0-t_, and *AUC*_0-∞_ increased with the dosage from 100 to 400 mg; median *T*_max_ was 0.5 h for 100 or 200 mg and 0.67 h for 400 mg dosage; geometric means (GM) of *t*_1/2_ were 0.80, 0.75, and 0.89 h for three dosages; GM of *MRT*_0-t_ or *MRT*_0-∞_ was comparable for the dosage of 100 or 200 mg, while that of 400 mg was significantly increased (*p* < 0.05); GM of plasma clearance (*CL/F*) or apparent volume of distribution (*Vd/F*) varied as 15.76–17.41 L/h or 18.12–22.46 L for the dosage of 100–400 mg, with no significant difference noted (*p* > 0.05). Linear pharmacokinetics among doses was evaluated and shown in Supplementary Material.

In addition, no gender effect on pharmacokinetics has been noticed. Geomean ratios (male/female) of ln*C*_max_, ln*AUC*_0-t_, and ln*AUC*_0-∞_ over the dosage of 100–400 mg were 0.98–1.01, 0.98–0.99, and 0.98–0.99, respectively. Gender also did not play a significant role in the variability of *T*_max_, *t*_1/2_, or *MRT*_0-t_.

The analytical method employed for pharmacokinetics was well validated according to the Food and Drug Administration guidance for validation of bioanalytical methods. The specificity, linearity, accuracies and precisions, matrix effects, extraction recovery, dilution integrity, stability of standard solution, or plasma samples were evaluated with detailed data shown in Supplementary Material. These results demonstrated that the established method for sample extraction, storage, and intermittent analysis was suitable for the high throughput sample analysis.

### Urinary Excretion

Urinary excretion parameters, such as urinary excretion rate, cumulative amount excreted, or percent of dose recovered in urine, as well as urine elimination rate constants were revealed in the current work (shown in Table 3).

**TABLE 3:** Urinary excretion parameter of TBPM after oral administration at dosage of 100, 200, and 400 mg.

	Time period (h)	TBPM-PI (100 mg, *n* = 12)	TBPM-PI (200 mg, *n* = 12)	TBPM-PI (400 mg, *n* = 12)
Urinary excretion rate (mg/h)	0–1	44.40 ± 15.15	75.90 ± 22.81	103.63 ± 45.45
	1–2	33.58 ± 9.29	87.24 ± 25.33	156.98 ± 34.88
	2–4	5.15 ± 2.30	10.99 ± 4.09	23.69 ± 10.76
	4–6	0.88 ± 0.50	2.06 ± 1.50	4.89 ± 3.75
	6–8	0.18 ± 0.09	0.39 ± 0.23	0.90 ± 0.48
	8–12	0.03 ± 0.01	0.06 ± 0.04	0.13 ± 0.07
	12–24	0.00 ± 0.00	0.00 ± 0.01	0.01 ± 0.01
Cumulative amount excreted in urine (mg)		90.52 ± 9.29	190.29 ± 23.74	320.18 ± 43.98
Percent of dose recovered in urine (%)		90.52 ± 9.29	95.14 ± 11.87	81.31 ± 11.36
Urine elimination rate constants (h^−1^)		0.70 ± 0.13	0.62 ± 0.20	0.49 ± 0.16

Note: Data were present as mean ± SD.

TBPM was excreted in urine rapidly after oral administration. The maximum urinary excretion rate was achieved at 0–1 or 1–2 h for treatment of 100 or 200–400 mg. The cumulative amount excreted in urine by 24 h was 90.52 ± 9.29, 190.29 ± 23.74, and 320.18 ± 43.98 mg for the dosage of 100, 200, and 400 mg, respectively. The percentage of administered dose recovered in urine was 90.52 ± 9.29%, 95.14 ± 11.87%, and 80.04 ± 10.99% (significantly lower than other doses, *p* < 0.05), respectively. Urine elimination rate constants were 0.70, 0.62, and 0.49 h^−1^ for three dosages, with a significant difference noticed between 100 and 400 mg (*p* < 0.05).

A gender effect on urinary excretion was also investigated. At dosage of 100 and 400 mg, ratios of percentage recovered in urine (male/female) were 0.96 and 0.93, respectively, with no significant gender difference observed (*p* > 0.05). However, a significant gender difference was noticed for the ratio of percentage recovered in urine for the dosage of 200 mg (male/female 0.83, *p* < 0.05). No significant gender differences were observed for urine elimination rate constants of 100 and 200 mg (male/female ratios 0.99 and 0.98, *p* > 0.05), except for dose of 400 mg (ratio 0.67, *p* < 0.05). The reason for the difference was complex and remains to be investigated.

The employed LC-MS/MS method for the determination of TBPM in urine was well validated as similar protocols for plasma samples. All validated items met the requirements of the Food and Drug Administration guidance for validation of bioanalytical methods and are shown in Supplementary Material.

### Pharmaco-Metabolomics

TOF-MS spectra of series time periods in urine samples were collected. After deconvolution, alignment, and filtration by the 80% rule, 2601 ions (mass-RT features) were obtained for each urine spectrum. PCA and OPLS-DA were conducted for the output data.

**FIGURE 3:**
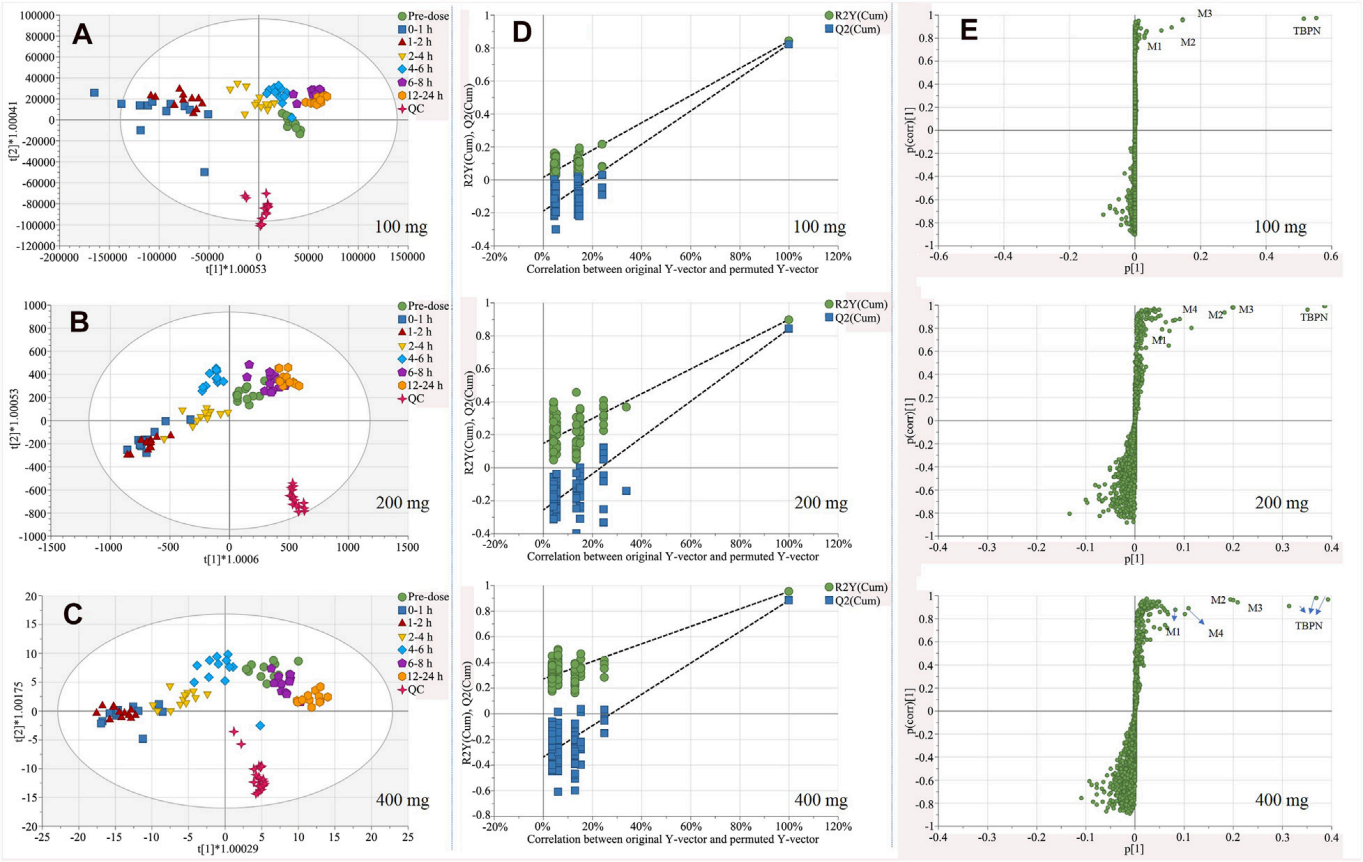
OPLS-DA score plot for urine samples of pre-dosedose, 0–1, 1–2, 2–4, 4–6, 6–8, and 12–24 h after oral administration of 100 mg **(A)**, 200 mg **(B)**, 400 mg **(C)** of TBPM-PI, and corresponding permutation tests **(D)**, as well as S-plots **(E)** of 0–1 h vs. per-dose urine samples in three dose group (*n* = 12 for each dose). Note: *t* (1) and *t* (2) indicate the first and the second components in the OPLS-DA model; R^2^Y (cum) means cumulative R^2^Y for the extracted components; Q^2^ (cum) was cumulative Q^2^ for the extracted components; p (1) indicates the importance of the variables in approximating X in the first component. p (corr) (1) shows the correlation coefficient of p (1) between X and *t*.

According to the PCA score plot, metabolic trajectory of perturbation and recovery was observed. To maximize the metabolism tendency, the OPLS-DA model was employed for each dose group. Corresponding OPLS-DA score plots for each dose group and S-plots of 0–1 h vs. pre-dose are shown in Figure 3. OPLS-DA models of 100, 200, or 400 mg were well fitted, with R^2^X 0.963, 0.811, or 0.842; *R* ^2^Y 0.565, 0.66, or 0.794; and Q^2^ 0.521, 0.593, or 0.636. The permutation test was conducted to validate models (shown in Figure 3D). All permuted *R* ^2^ and Q^2^ values on the left were lower than those of the original point on the right, indicating good stability and credibility.

According to the score plot obtained (Figures 3A–C), regression tendency was noticed: maximum metabolism interruption was observed at 0–1 h (100 mg) or 1–2 h (200, 400 mg); subsequently, urine samples of following time periods regressed back to pre-dose samples; restoration of metabolism profiles was achieved by 6–8 h (100 mg) or 12–24 h (200, 400 mg). The regression tendency illustrated metabolic interruption and elimination of TBPM *in vivo*.

**FIGURE 4:**
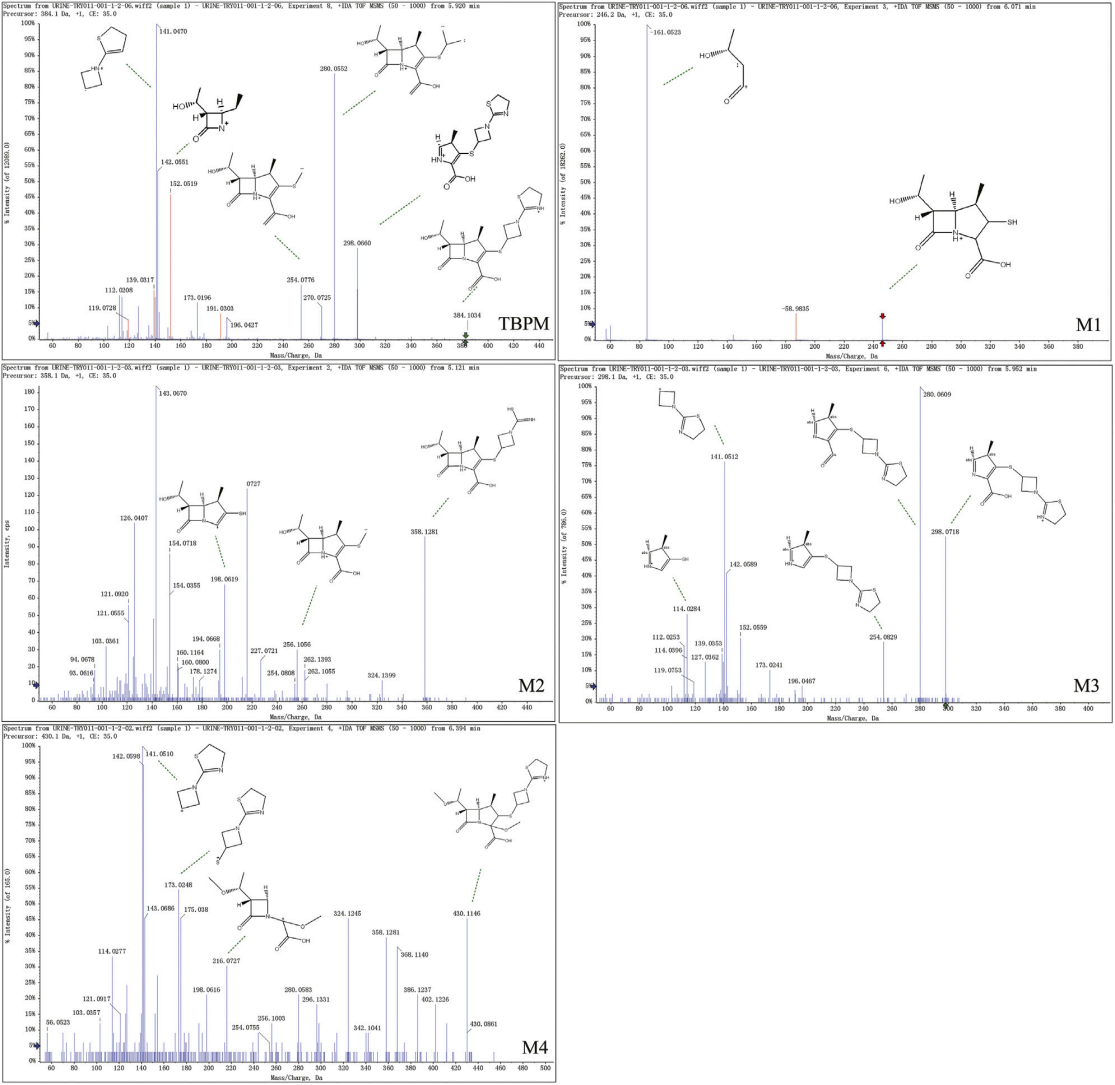
The MS^2^ spectrum of TBPM, M1, M2, M3, M4, and fragment ions in the positive ion mode.

**FIGURE 5:**
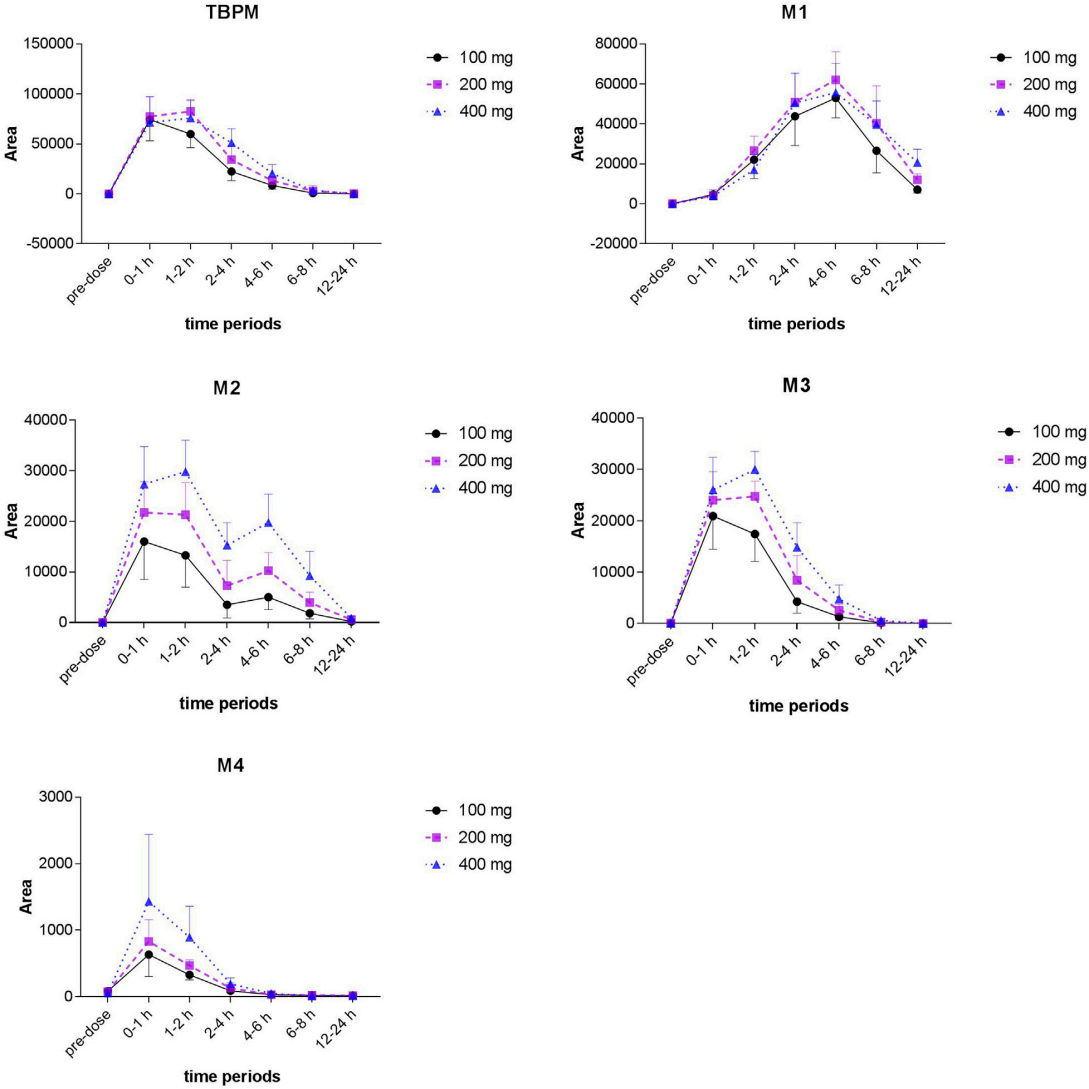
Area–time profiles of TBPM, M1 to M4 detected in urine after a single oral dose of 100, 200, or 400 mg in healthy Chinese subjects (*n* = 12 for each dose).

From S-plot of pre-dose and 0–1 h urine samples (Figure 3E), TBPM and tentatively identified metabolites (M1–M4) were identified. The MS^2^ spectra of TBPM and metabolites are present, as shown in Figure 4. MASS feature, formula, mass deviation, RT, fragments of TBPM, and metabolites are listed in Table 4. According to abundance of TBPM, M1 to M4 in the urine metabolomic profile, the area–time profiles are drawn in Figure 5. The maximum urine area of TBPM or M3 was found at 0–1 h (dose of 100 mg) or 1–2 (dose 200–400 mg) h after TBPM-PI treatment; almost complete elimination was achieved by 12–24 h. For M1, the maximum urine area was observed at 4–6 h, and the excretion curve was not drawn back to the pre-dose level by 12–24 h; for M2, a double peak was detected, which remains to be explained; for M4, the maximum urine area was detected at 0–1 h, and vanishment was achieved at 12–24 h.

**TABLE 4:** Identified metabolites of TBPM in urine after oral administration.

Id	Metabolite description	Formula	RT (min)	m/z	Neutral mass	Mass deviation (ppm)	Fragment ions	VIP
TBPM	Parent [M + H]^+^	C_16_H_21_N_3_O_4_S_2_	5.93	384.1030	383.0957	0.8	298.0660, 280.0552, 254.0776, 142.0551, and 141.0470	28.0, 19.0, 18.1
M1	Loss of 137.9361 [M + H]^+^	C_10_H_15_NO_4_S	6.15	246.1685	245.1613	0.8	187.1004, and 85.0315	1.7, 4.2, 4.1
M2	Loss of 25.9798 [M + H]^+^	C_14_H_19_N_3_O_4_S_2_	5.16	358.1249	357.1176	0.8	324.1415, 256.1056, 198.0619, 194.1029, 103.0353	5.8, 9.2, 9.5
M3	Loss of 86.0380 [M + H]^+^	C_15_H_11_N_3_O_2_S	5.93	298.0667	297.0594	1.0	280.0609, 254.0829, 141.0512, and 114.0284	7.4, 10.1, 9.8
M4	Gain of 46.0053 [M + H]^+^	C_17_H_23_N_3_O_6_S_2_	6.38	430.1077	429.1027	0.7	412.1146, 402.1226, 216.0727, 173.0248, and 141.0510	1.4, 4.6, 4.9

Note: VIP value were from OPLS-DA models of 0–1 h versus pre-dose samples at 100, 200, and 400 mg.

### Safety Profile

There were no serious AEs, and no subjects withdrew from the trial due to AEs. Among all subjects receiving TBPM-PI, 16 experienced a total of 20 AEs (Table 5), including six cases of six individuals (50.0%) in the 100 mg group, six cases of five individuals (41.7%) in the 200 mg group, and eight cases of five individuals (41.7%) in the 400 mg group. A total of 13 AEs in 12 subjects were judged as possibly related to TBPM-PI treatment, involving four cases of hyperuricemia (each one in 100 or 200 mg dose, two in 400 mg dose, 11.1%), four cases of ECG abnormality (each one in 100 or 200 mg dose, two in 400 mg dose, 11.1%), one case of nausea (one in 400 mg dose, 2.8%), one case of dizziness (one in 400 mg dose, 2.8%), one case of increased blood bilirubin (one in 200 mg dose, 2.8%), one case of decreased blood pressure (one in 100 mg dose, 2.8%), and one case of mouth ulcer (one in 200 mg dose, 2.8%). All AEs were mild, and all subjects experiencing AEs recovered without any treatment or intervention, except for six cases who were lost to follow-up.

**TABLE 5:** Summary of AEs for all treated subjects.

	No. (%) of subjects (no. of events)		
	100 mg (*n* = 12)	200 mg (*n* = 12)	400 mg (*n* = 12)
AEs	6 (50.0) **(**6**)**	5 (41.7) **(**6**)**	5 (41.7) **(**8**)**
Drug-related AEs	3 (25.0) **(**3**)**	4 (33.3) **(**4**)**	5 (41.7) **(**6**)**
Hyperuricemia	1 (8.3) **(**1**)**	1 (8.3) **(**1**)**	2 (16.7) **(**2**)**
Abnormal ECG			
Sinus arrhythmia	—	1 (8.3) **(**1**)**	—
ST-T changes	1 (8.3) **(**1**)**	—	—
Electrocardiogram T wave abnormal	1 (8.3) **(**1)*	—	—
Sinus bradycardia	1 (8.3) **(**1)*	—	1 (8.3) **(**1**)**
Sinus bradycardia and sinus arrhythmia	—	—	1 (8.3) **(**1**)**
Decrease in blood pressure	1 (8.3) **(**1**)**	—	—
Increase in blood bilirubin	—	1 (8.3) **(**1**)**	—
Increase in urine leukocyte	1 (8.3) **(**1)*	1 (8.3) **(**1)*	1 (8.3) **(**1)*
Increase in urine erythrocyte	—	—	1 (8.3) **(**1**)**#
Nausea	—	—	1 (8.3) **(**1**)**
Dizziness	—	—	1 (8.3) **(**1**)**
Mouth ulcer	—	1 (8.3) **(**1**)**	—
Anemia	—	1 (8.3) **(**1)*	—

Note: * or # indicate AE relationship with drugs of not related or unclear.

## Discussion

### Pharmacokinetics

Limited studies aiming at exploring the pharmacokinetics of TBPM have been documented (Eckburg et al., 2019; Nakashima, et al., 2009b; Xue, et al., 2019). These studies provided pharmacokinetic parameters of TBPM either in a single dose or in single gender. As a result, dose or gender effects on pharmacokinetics of TBPM have not been investigated. The current work investigated dose and gender effects on pharmacokinetics of TBPM in the Chinese population for the first time.

The pharmacokinetic profiles revealed in the current work were consistent with those of the previous Japanese reports for parameter values of *T*_max_, *t*_1/2_, and dose-proportional manners of *C*_max_ and *AUC*_0-∞_ (Nakashima, et al., 2009b). However, our work revealed 1.2–1.6 fold up of *C*_max_ or *AUC*_0-∞_ in healthy Chinese volunteers compared with corresponding data in Japanese volunteers at designed dosage levels. The difference may be attributed to ethnic differences among the Japanese and Chinese population, and may lead to modulation of dosage in the Chinese clinical use.

The current work reproduced parameters of *T*_max_, *C*_max_, *AUC*_0-∞_, and *MRT*_0-t_ previously reported in healthy Chinese or Australian subjects (Eckburg, et al., 2019; Xue, et al., 2019). However, inconsistencies were also noticed; although *t*_1/2_ in our study was close to that in previous report in Chinese volunteers (Xue, et al., 2019), it was observed as 1.0 h in Australian volunteers (Eckburg, et al., 2019). For *CL/F* and *Vd/F*, our work revealed a 16–24% or 25–43% decrease compared with that in healthy Australian subjects (Eckburg, et al., 2019). The inconsistence may be attributed to ethnic, formulation, or other factors.

The dose effect was evaluated in the current work. It was noted that *T*_max_ of 400 mg was significantly delayed compared with that of 100 mg (0.68 vs. 0.50 h and *p* < 0.05), consistent with the previous report (Nakashima, et al., 2009a). The delay may be attributed to the saturation of intestinal influx transporters (mainly OATP 1B1 and 2B1) (Kato, et al., 2010). In agreement with the previous report in Japanese volunteers, linearity was observed for *C*_max_ over dose ranges of 100–200 mg and *AUC*_0-∞_ over dose ranges of 100–400 mg. The proportionality coefficient (β) for *C*_max_, *AUC*_0-t_, and *AUC*_0-∞_ was 0.77, 0.93, and 0.93, respectively (Supplementary Material). The power model analysis indicated that the lower and upper limits of the 90% CI for β almost overlapped acceptance intervals for *AUC*_0-t_ and *AUC*_0-∞_, while were not within acceptance intervals for *C*_max_. Moreover, the current work revealed *CL/F* of 15.93–17.53 L/h and *Vd/F* of 17.71–23.35 L for doses of 100–400 mg, and no significant difference among doses was noted (*p* > 0.05). For *MRT*_0-t_, it was shown that *MRT*_0-t_ of 400 mg was significantly increased compared with that of 100 or 200 mg (*p* < 0.05), indicating prolonged residence *in vivo* at a dose of 400 mg. The extension may be explained by saturation of intestinal influx transporters (Kato, et al., 2010).

To our knowledge, no gender effect on pharmacokinetics of TPBM has been investigated in previous studies. Our work revealed no significant gender differences in parameters of *C*_max_, *AUC*_0-t_, *AUC*_0-∞_, *T*_max_, *t*_1/2_, and *MRT*_0-t_.

Additionally, large intersubject variability in *C*_max_, *t*_1/2_, or *Vd/F* was observed in both the current work and previous reports, which may be explained by variability in absorption or prodrug conversion among subjects (Eckburg, et al., 2019; Nakashima, et al., 2009b; Xue, et al., 2019).

### Urinary Excretion

TBPM-PI was quickly absorbed and converted to TBPM, which was distributed into the kidney and eliminated through urinary excretion. It has been reported that about 60.7–67.7 or 59.2% of TBPM was eliminated from urine by 24 h in Japanese (Nakashima, et al., 2009b) or Australian volunteers (Eckburg, et al., 2019). Our work was the first report to reveal urinary excretion of TBPM in Chinese volunteers.

In contrast to the percentage of dose recovered in urine by 24 h in Japanese volunteers (Nakashima, et al., 2009b), it seems that the percentage of dose recovered in urine was higher in Chinese volunteers. The discrepancy was worth further investigating and may be attributed to ethnic difference.

For the dose effect, it was noted that the percentage of dose recovered in urine for 400 mg was significantly lower than that of 100 or 200 mg (*p* < 0.05). A similar tendency was also observed in the previous report (Nakashima, et al., 2009b). The decrease may be explained by variation in the ratio of metabolites or excretion (Nakashima, et al., 2009b). In addition, the decrease may be explained by decreasing urine elimination rate constants, of which a significant difference was noticed between 100 and 400 mg (0.70 vs. 0.49 h^−1^).

Gender effect on urinary excretion was also investigated. However, the effect was complex and remains to be investigated. For dosage of 100 mg, no gender difference was noticed for percentage recovered in urine or urine elimination rate constants. For dosage of 200 mg, a significant gender difference was noticed for the percentage recovered in urine (male/female 0.83, *p* < 0.05). However, no significant gender difference in percentage recovered in urine was observed for dosage of 400 mg, which may be offset by significant decreased urine elimination rate constants of 400 mg. In addition, a significant gender difference was observed for urine elimination rate constants at dosage of 400 mg (male/female 0.67, *p* < 0.05).

### Pharmaco-Metabolomics

Pharmacokinetic study aims at pharmacokinetic features. Pharmaco-metabolomics aims at investigating relationship between metabolomic profiles and biologic effect of drugs (Everett 2016). Pharmaco-metabolomics can be an extension of pharmacokinetics, and shall reveal metabolites of drugs, biomarkers related with drug efficacy, or adverse effect in the pharmacokinetic study. To our knowledge, pharmaco-metabolomics has been seldom introduced in the pharmacokinetic study in healthy volunteers.

The current work conducted pharmaco-metabolomic research based on a pharmacokinetic study. Maximum metabolism interruption observed was consistent with the maximum urinary excretion rate of TBPM observed at 0–1 h (100 mg) or 1–2 h (200 and 400 mg). Subsequent regression could be explained by restoration of metabolism profiles and the decreasing urinary excretion rate. The current work for the first time provided evidence that urine metabolism interruption of TBPM was recovered by 12–24 h, proving elimination of drugs and recovery of the metabolic state in the pharmacokinetic study.

**FIGURE 6:**
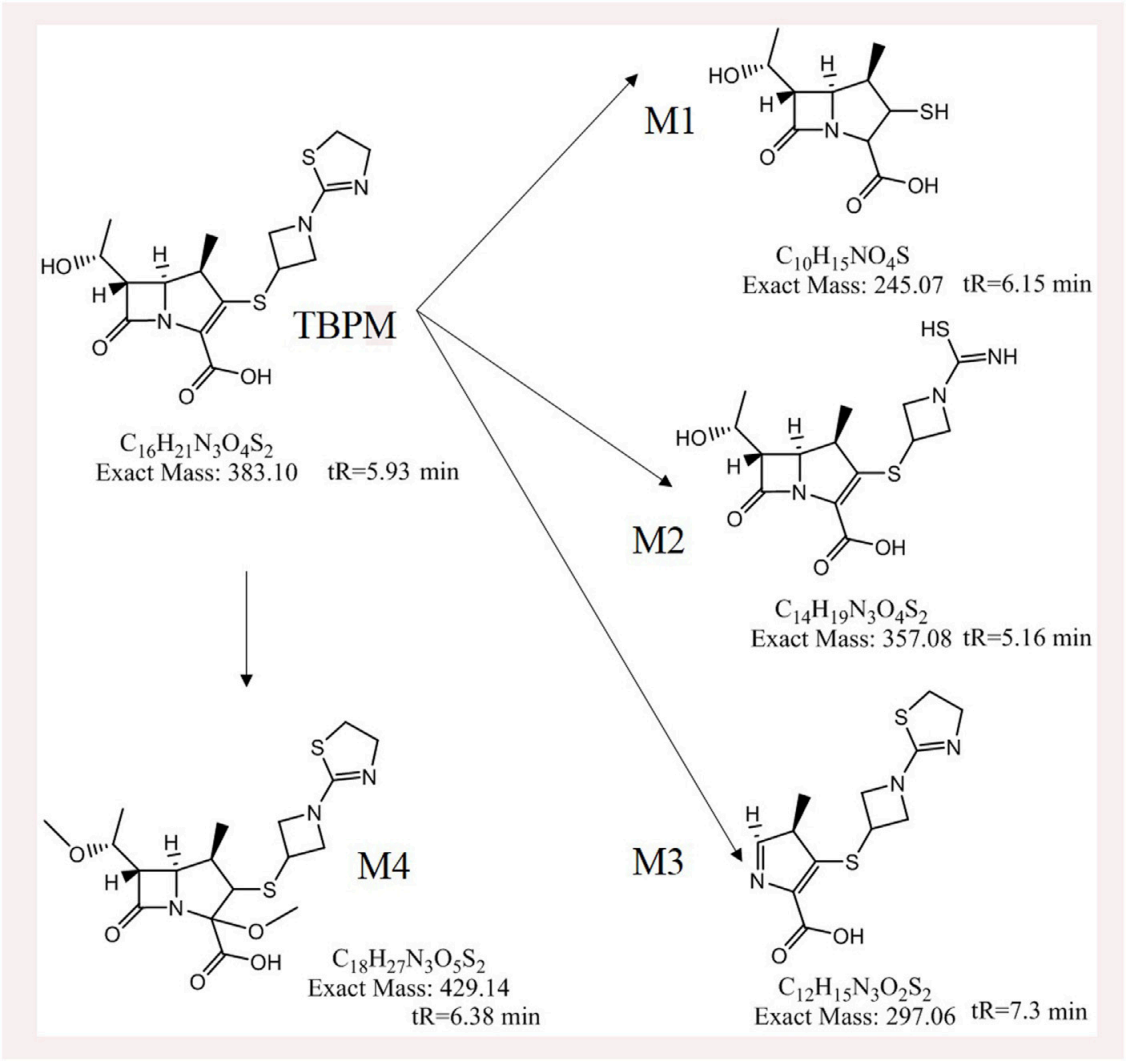
Proposed metabolic pathways of TBPM to M1–M4.

Urine metabolite information of TBPM was also investigated. The metabolic pathways of TBPM-PI were proposed (Figure 6). First, TBPM-PI was de-pivoxil to yield the active metabolite TBPM (m/z 384.1030, RT 5.93 min) (Kato, et al., 2010). Subsequently, a group of 2-(3-(l1-sulfanyl)azetidin-1-yl)-4,5-dihydrothiazole was degraded from TBPM to produce M1, with extract mass of 246.1685 and RT of 6.15 min. M2 was produced by the cycloreversion reaction of dihydrothiazole from TBPM, with m/z of 358.1249 and RT of 5.16 min. M3 was a product obtained by the removal of *β*-lactam ring, with m/z of 298.0667 and RT of 5.93 min. M4 was formed by the addition of methoxyl and methyl groups, with m/z of 430.11 and RT of 6.38 min. A difference was noticed compared with the previous report in Japanese volunteers, where opened ring metabolites (such as LJC11, 562) were observed in urine (Nakashima, et al., 2009a). However, no opened ring metabolite was noticed in urine of Chinese population, which may indicate ethnic difference in the metabolism of TBPM. Human dehydropeptidase-1 (DHP), the opened β-lactam ring metabolite–dependent hydrolase, may be differently expressed between the Chinese and Japanese population.

Area–time profiles of TBPM obtained were consistent with the urinary excretion rate observed. In addition, the tendency was confirmed by deviation and regression in the pharmaco-metabolomic study. For metabolites from M1 to M4, however, the current work fails to conduct absolute quantification for short of standard reference. As a result, detailed urine excretion and pharmacokinetic characteristics of metabolites remain to be revealed in the future study.

Another limitation of the current work was the absence of the metabolomic analysis of plasma samples. Limited by authority, blood samples were not approved for the metabolomic analysis in the current work, failing to reveal metabolomic intervention and metabolites in the plasma. However, pharmaco-metabolomics of urine samples exhibits noninvasive advantage and was easy to carry out in the clinical study.

### Safety

Previously, it was reported that gastrointestinal disorders (primarily diarrhea) were ranked the most common type of AEs caused by TBPM-PI treatment (ME1211-15/16, data not published). Diarrhea was not observed in the current research, while nausea or dizziness was observed at dosage of 400 mg (1/12) with a similar incidence in the previous work (ME1211-14, not published). Inconsistently, hyperuricemia (4/36) was frequently observed in the current work, requiring more attention in the clinical use in the Chinese population. Besides, abnormal ECG was noticed in the current work. Nevertheless, all AEs were mild in the current work. No serious AEs or withdrawal due to AEs was found. Generally, this study confirmed the safety and tolerance of single-dose administration of oral TBPM-PI over dose of 100–400 mg in Chinese healthy volunteers.

## Conclusion

In summary, this was the first pharmacokinetics, urine excretion, and pharmaco-metabolomics of TBPM-PI after single escalating oral doses in healthy Chinese volunteers, bridging corresponding information with previous completed clinical trials in Japan. The safety and PK behaviors in the current clinical trial supported further phase II clinical trials in pediatric infection in China. The pharmaco-metabolomic analysis revealed metabolic interruption and elimination of TBPM in healthy volunteers, providing information other than pharmacokinetics in the pharmacokinetic study. Pharmaco-metabolomics employed in the current work deserves to be followed in other pharmacokinetic studies.

## Data Availability

The datasets presented in this article are not readily available because they belong to Jiangsu Carephar Pharmaceutical Co., Ltd and the First Affiliated Hospital of Zhengzhou University. Requests to access the datasets should be directed to Xin Tian, tianx@zzu.edu.cn.
