# Supplementary material for: Pharmacokinetics, Urinary Excretion, and Pharmaco-Metabolomic Study of Tebipenem Pivoxil Granules After Single Escalating Oral Dose in Healthy Chinese Volunteers
Source: Front Pharmacol. 2021 Jul 13;12:696165. doi: 10.3389/fphar.2021.696165 (PMC8314177; doi:10.3389/fphar.2021.696165)
Supplement: Supplementary file 1 [file Table1.DOC]

**Validation of quantification method for TBPM in plasma or urine samples**

The analytical method was validated according to Food and Drug Administration guidance for validation of bioanalytical methods. The specificity was demonstrated by comparing the MRM chromatograms of blank samples from 6 six individual volunteers with those of spiked standard samples. No endogenous substances interfere target peaks. Good linearity was obtained over the range of 20.0-20000 ng/mL, with correlation coefficient (*r*) of 0.998. The lower limit of quantification (LLOQ), quality control of low (LOQ), middle (MOQ) and high (HOQ) concentrations were set at 20.0, 60.0, 2500, and 15000 ng/mL. The intra-run (*n*=6) accuracies and precisions were evaluated as relative error (RE) of -7.1%-2.3% and coefficient of variation (CV) of 5.5% - 9.3% for LLOQ, RE of -4.4% -7.1% and 2.0%-4.1% for QC samples, indicating good accuracy and precision of the method. In the inter-run investigation, the RE and CV in same concentration from three analytical batches were both less than 8.0%, indicating good repeatability between runs. The CVs of the IS-normalized matrix effects at LQC, HQC samples were less than 2.3%, suggesting that the ionization of TBPM was not disturbed by the human plasma matrix. The extraction recovery was 99.2% for TBPM at QC levels (CV of 6.0%) and 98.4% for IS (CV 1.8%), confirming good reliability of the method. Dilution integrity of 5 times was evaluated with RE 1.4% and RSD 4.4%, maintaining accuracy and precision of testing for high concentrations samples. Stability of [standard](javascript:;) [solution](javascript:;) at room temperature or 4 °C, plasma samples after five freeze-thaw cycles at -80 °C or room temperature, post-extraction solution at room temperature, analyte in matrix after long term [storage](javascript:;) under -20°C or -80°C were evaluated, with all CVs and REs meeting the requirements. These results demonstrated that the established method for sample extraction, storage, and intermittent analysis was suitable for high throughput sample analysis.

The LC-MS/MS method developed for determination of TBPM in urine was well validated as similar protocols for plasma samples. The Linearity range was 100-50000 ng/mL, with LLOQ, LQC, MQC, and HQC of 100, 300, 2500 and 37500 ng/mL. The RE and CV inter-run (*n* = 6) or intra-run (*n* = 6) ranged over -7.9%-9.3% and 1.4% -5.0% for LLQC, LQC, MQC and HQC. The CVs of matrix effects were less than 2.3%. The extraction recovery varied from 97.5%-99.7% and 97.4% for TBPM and IS in urine. Dilution integrity of 100 times was evaluated with RE and RSD of -11.5% and 0.6%. Stability for [standard](javascript:;) [solution](javascript:;) at room temperature or 4°C, urine samples after four freeze-thaw cycles at -80 °C or room temperature, post-extraction solution at room temperature, analyte in matrix after long term [storage](javascript:;) under -20°C or -80°C were evaluated, demonstrating that the established method was suitable for large scale sample analysis. All the validated items met the requirements of Food and Drug Administration guidance for validation of bioanalytical methods.

Table Linear relationship between main pharmacokinetic parameters and dose

| **Dependent** | **R^2^** | **Intercept**  **α** | **Slope**  **β** | **p** | **90% CI of β** | **discrimination intervals** |
| --- | --- | --- | --- | --- | --- | --- |
| **AUC_0-∞_** | 0.9343 | 4.4878 | 0.9284 | <0.0001 | 0.8426-1.0142 | 0.9255-1.0745 |
| **AUC_0-t_** | 0.9340 | 4.4654 | 0.9319 | <0.0001 | 0.8456-1.0183 | 0.9255-1.0745 |
| C_max_ | 0.7161 | 5.1655 | 0.7684 | <0.0001 | 0.5998-0.9370 | 0.8809-1.1194 |
